# Transition to end-of-life care in patients with neurological diseases in an acute hospital ward

**DOI:** 10.1186/s12883-024-03768-z

**Published:** 2024-07-22

**Authors:** Gudrun Jonsdottir, Erna Haraldsdottir, Runar Vilhjalmsson, Valgerdur Sigurdardottir, Haukur Hjaltason, Marianne Elisabeth Klinke, Gudny Bergthora Tryggvadottir, Helga Jonsdottir

**Affiliations:** 1 Faculty of Nursing and Midwifery, School of Health Sciences, University of Iceland, Reykjavik, 101 Iceland; 2 Department of Hematology and Oncology, Landspitali, The National University Hospital of Iceland, Reykjavik, 101 Iceland; 3 Division of Nursing and Paramedic Science, Queen Margaret University, Edinburgh, EH216UU Scotland; 4 Palliative Care Unit, Landspitali, The National University Hospital of Iceland, Reykjavik, 101 Iceland; 5 Faculty of Medicine, School of Health Sciences, University of Iceland, Reykjavik, 101 Iceland; 6 Department of Neurology, Landspitali, The National University Hospital of Iceland, Reykjavik, 101 Iceland; 7 The Directorate of Health, Katrinartun 2, Reykjavik, 105 Iceland; 8 Respiratory Section, Division of Clinical Services, Landspitali, The National University Hospital of Iceland, Reykjavik, 101 Iceland

**Keywords:** Neurodegenerative diseases, Stroke, ALS, Parkinson’s disease, Terminal care, Length of stay, Acute hospital ward, Tertiary hospital, Hospitalisation

## Abstract

**Background:**

Transitioning to end-of-life care and thereby changing the focus of treatment directives from life-sustaining treatment to comfort care is important for neurological patients in advanced stages. Late transition to end-of-life care for neurological patients has been described previously.

**Objective:**

To investigate whether previous treatment directives, primary medical diagnoses, and demographic factors predict the transition to end-of-life care and time to eventual death in patients with neurological diseases in an acute hospital setting.

**Method:**

All consecutive health records of patients diagnosed with stroke, amyotrophic lateral sclerosis (ALS), and Parkinson’s disease or other extrapyramidal diseases (PDoed), who died in an acute neurological ward between January 2011 and August 2020 were retrieved retrospectively. Descriptive statistics and multivariate Cox regression were used to examine the timing of treatment directives and death in relation to medical diagnosis, age, gender, and marital status.

**Results:**

A total of 271 records were involved in the analysis. Patients in all diagnostic categories had a treatment directive for end-of-life care, with patients with haemorrhagic stroke having the highest (92%) and patients with PDoed the lowest (73%) proportion. Cox regression identified that the likelihood of end-of-life care decision-making was related to advancing age (HR = 1.02, 95% CI: 1.007–1.039, *P* = 0.005), ischaemic stroke (HR = 1.64, 95% CI: 1.034–2.618, *P* = 0.036) and haemorrhagic stroke (HR = 2.04, 95% CI: 1.219–3.423, *P* = 0.007) diagnoses. End-of-life care decision occurred from four to twenty-two days after hospital admission. The time from end-of-life care decision to death was a median of two days. Treatment directives, demographic factors, and diagnostic categories did not increase the likelihood of death following an end-of-life care decision.

**Conclusions:**

Results show not only that neurological patients transit late to end-of-life care but that the timeframe of the decision differs between patients with acute neurological diseases and those with progressive neurological diseases, highlighting the particular significance of the short timeframe of patients with the progressive neurological diseases ALS and PDoed. Different trajectories of patients with neurological diseases at end-of-life should be further explored and clinical guidelines expanded to embrace the high diversity in neurological patients.

## Introduction

End-of-life care generally refers to care provided in the last days and weeks of life [1, 2], although the timeframe may range up to a year [3]. The decision to transfer patients to end-of-life care entails shifting the focus from life-sustaining treatment to emphasizing provision of comfort care based on individual patients’ needs. As a part of palliative care, end-of-life care decision-making is a complex process based on comprehensive consideration of patients’ deteriorating health, including indications of physical, social, spiritual, and psychological decline towards death [1]. End-of-life care is delivered in collaboration with interdisciplinary team and with patients and next-of-kin. The goal of end-of-life care is to relieve suffering, including relieving pain and other distressing symptoms, and improve the quality of living and dying by providing psychological and social support to manage physical, emotional, social, and spiritual burden of the imminent death [4].

The realisation of the end-of-life stage in patients with neurological diseases may be difficult due to fluctuating course of diseases, complex presentations of symptoms, and variations in prognosis [1]. Realising the proper transition time for end-of-life care is therefore important [2–7]. Evidence-based general clinical guidelines for end-of-life care exist [4, 8, 9], and specific guidelines for patients with neurological diseases are emerging [3, 5, 6]; however, there is a common notion that the period of end-of-life care for patients is too short [10–13]. An exception to this are patients with cancer, for which the time from transition to end-of-life care to death is longer and has a more predictable trajectory than for patients with many other incurable diseases [10–13]. Of particular concern is the late transition to end-of-life care within acute wards in hospitals, which is often made at too advanced stages [11]. Uncertainty in the disease trajectory for non-cancer patients and bringing together patients requiring life-sustaining treatment and end-of-life care in the same acute care ward, as well as rigid practice habits, might contribute to this late transition [14]. Being aware of patients’ preferences can be hampered due to a lack of conversations with patients, although they might still be capable of communicating effectively, resulting in decision-making being deferred to relatives [15–21]. Accentuating this is the distinct presentation of neurological diseases, specifically ischaemic and haemorrhagic stroke, amyotrophic lateral sclerosis (ALS), and Parkinson’s disease and other extrapyramidal diseases (PDoed), which frequently present with cognitive, physical, and mental impairments, often leading to unpredictable and variable disease progression [1, 3, 15, 22–30]. Signs and symptoms of these impairments [16, 28, 31–33], which can occur years before death, may resemble those of dying, such as pain, dyspnoea, fatigue, anxiety, loss of appetite, dysphagia, increased dependency, loss of consciousness, loss of mobility, communication difficulties, and cognitive dysfunction [34–36].

Patients with haemorrhagic stroke exhibit higher mortality rates than patients with ischaemic stroke, with a greater likelihood of mortality occurring within days or weeks after admittance to a hospital [37–40]. In some studies, the time from ischaemic stroke diagnosis to death was three days [41, 42]. In a study of patients with PDoed, the timeframe from change in treatment directive until death was 0 days [43]. Comparable studies of patients with ALS were not retrieved. Failure to recognise neurological patients as dying can lead to inappropriate treatments and interventions [44, 45]. It can hinder quality end-of-life care and needed symptom control [46], and cause distress for patients, relatives, and healthcare professionals [21, 46–49]. Examining what may influence timely transition to end-of-life care for patients with neurological diseases is pertinent to better understand the care decision-making process [50, 51]. This study aimed to analyse the timeframe of end-of-life care decision-making and death in an acute neurological hospital ward. More specifically, we aimed to identify whether and how previous treatment directives, medical diagnoses, and demographic factors predict the duration of stay in an acute hospital ward until end-of-life care decision-making and death, respectively.

## Materials and methods

In this retrospective study, data were retrieved from patient health records (PHRs) used in routine daily practice of adult patients who died in an acute neurological hospital ward in the Landspitali National University Hospital of Iceland. The ward specialises in serving patients with neurological diseases and is the only ward in the country of this kind. The ward serves as a tertiary hospital for Reykjavik capital area and at times provides general medical neurology care in parallel to highly specialised medical care.

Data were collected from 1st January 2011 to 31st August 2020. Four trained data abstractors extracted data of the following variables from the PHR: Age, gender, medical diagnosis, duration of stay (number of hospital days) from admission until the transition to end-of-life care, length of time from transition to end-of-life care to death during the patients’ last admission, and admittances in the last year of life. The decision of transition to end-of-life care is documented by the physician in the PHRs. It reflects patients’ wishes and preferences for end-of-life care, such as whether they desire life-sustaining treatments or prefer comfort measures only [7]. It is also documented under a particular rubric on the PHRs’ front page. This study is part of a larger study investigating the care process near the end-of-life of patients with neurological diseases in an acute hospital ward. For collecting the data, the authors developed and tested the data collection tool NEOLCAT [52].

### Ethics approval

The Ethics Committee at Landspitali National University Hospital approved the study (Nr. 26/2017) with additional authorisation for data collection from 1st January 2017 to 31st August 2020, and renewed approval in July 2023 (Nr. 36/2023). No identifiable patient information was used, and thus, informed consent was not required.

### Participants

**Fig. 1:**
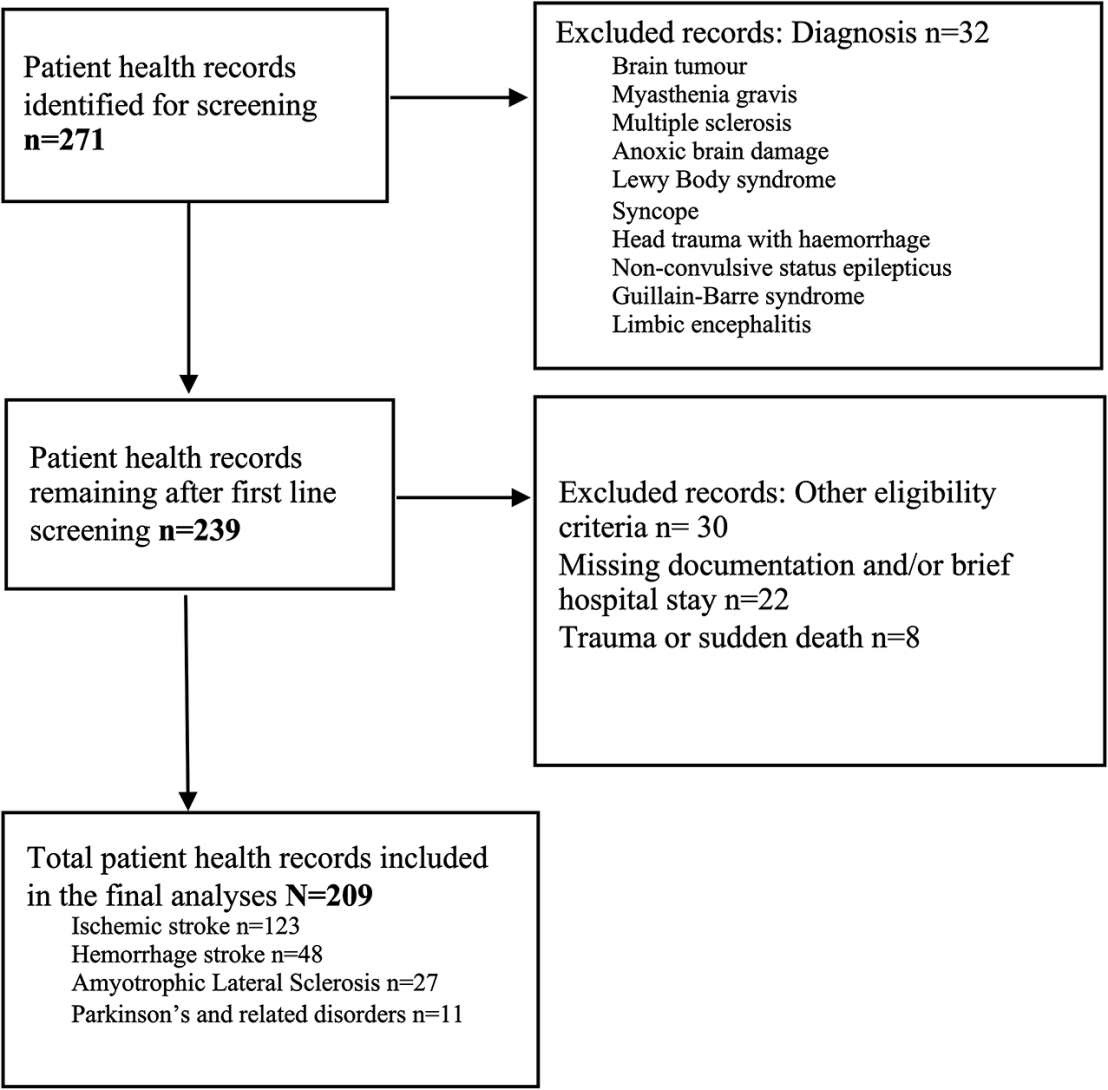
Flow chart of exclusion of patients’ health records

Patient health records of all patients (*n* = 271) who died at the neurological ward with a neurological disease diagnosis were screened. Those with the following ICD-10 codes were included in the study: I60-I64 stroke diagnoses, G12.2 amyotrophic lateral sclerosis (ALS), G20 Parkinson’s disease, and G25 extrapyramidal and movement disorders (PDoed). Excluded from the study were patients who belonged to patient groups to which only a few patients fitted, had a brief hospital stay (six days or shorter) or there was limited documentation of them in the PHRs (see Fig. 1).Thirty-two patients with brain tumour, myasthenia gravis, multiple sclerosis, anoxic brain damage, non-convulsive status epilepticus, Lewy Body syndrome, syncope, head trauma with haemorrhage, Guillain-Barre syndrome, and limbic encephalitis were excluded because of limited numbers of patients in those groups. Additionally, thirty patients were excluded due to; (a) 18 patients with limited documentation in the PHRs and (b) eight patients had a sudden death, and (c) there were four patients to whom both (a) and (b) applied. The medical diagnoses of those patients were ischaemic stroke (*n* = 10/30), haemorrhage stroke (*n* = 15/30), and ALS (*n* = 5/30). In total, 209 patients were included in the analysis. All patients were 18 years or older.

### Data analysis

Statistical analyses were conducted using the IBM Statistical Package for the Social Sciences (SPSS) [53]. The results of descriptive statistics are shown with descriptive bar graphs. In addition, multivariate analysis with Cox regression was used to see if treatment directives, medical diagnosis, age, gender, and marital status were related to the time until the decision of end-of-life care and the subsequent time until death. Differences were considered statistically significant if *p* < 0.05.

## Results

The majority of patients had ischaemic (*n* = 123) or haemorrhagic stroke (*n* = 48), followed by ALS (*n* = 27). PDoed (*n* = 11) comprised the smallest patient group (see Table 1). The average age of the patients was 78 years, and more than half of them were women. In the ischaemic stroke group, women comprised 57% (*n* = 70) of the patients, while men were the majority in the haemorrhagic stroke (*n* = 27, 56%), ALS (*n* = 15, 56%), and PDoed (*n* = 8, 73%) groups. Patients with ischaemic stroke were the oldest, with an average age of 81 years, while patients with haemorrhagic stroke were slightly younger, with an average age of 78 years, compared to 71 and 75 years, respectively, for patients with ALS and PDoed.

**Table 1:** Patient characteristics and patient hospital data (N = 209)

	Stroke-ischaemic (n = 123)	Stroke-haemorrhagic (n = 48)	ALS (n = 27)	PDoed (n = 11)	Total (N = 209)
	n (%)	n (%)	n (%)	n (%)	n (%)
Women	70 (57)	21 (44)	12 (44)	3 (27)	106 (51)
Men	53 (43)	27 (56)	15 (56)	8 (73)	103 (49)
Married, cohabiting	51 (46)	26 (60)	15 (65)	10 (91)	104 (55)
Single, widowed, divorced, etc.	59 (54)	17 (40)	9 (35)	1 (9)	86 (45)
Treatment directive for end-of-life care in the PHR	108 (88)	44 (92)	21 (78)	8 (73)	181 (87)
	**M (SD)**	**M (** **SD)**	**M (SD)**	**M (SD)**	**M (SD)**
Age in years at death	81 (10.3)	78 (12.1)	71 (9.3)	75 (12.2)	79 (11.3)
Hospital days in the last year	20 (26.9)	23 (22.2)	55 (45.1)	42 (26.1)	27 (31.1)
Hospital days during the last admission	15 (19.2)	15 (19.0)	30 (41.4)	27 (18.9)	18 (23.7)
Admittances in the last year (all hospital wards)	1.46 (1.3)	1.98 (1.6)	2.78 (1.5)	2.00 (1.1)	1.78 (1.3)

Fifty-two patients had a documented decision on end-of-life care after two days in the hospital ward. An additional 54 patients had a documented decision within three to five days, and 28 more patients had a documented decision within six to 11 days of admission. For another 63 patients, the decision on end-of-life care was made between 12 and 170 days of admission. Although the majority of the patients had a treatment directive for end-of-life care before death, the frequency differed among the patient groups: 88% (*n* = 108) of patients with ischaemic stroke, 92% (*n* = 44) of patients with haemorrhagic stroke, 78% (*n* = 21) of patients with ALS, and 73% (*n* = 8) of patients with PDoed (see Table 1). Twenty-eight, or 13% of the patients, were without a treatment directive, of whom there were 15 patients with ischaemic stroke, four patients with haemorrhagic stroke, six patients with ALS, and three patients with PDoed.

The length of hospital stay before death differed between the patient groups. Specifically, patients with ALS and PDoed stayed in the hospital longer before death than patients with stroke. The average stay of patients with ALS and PDoed was 55 and 42 days, respectively, compared with the 20–23-day stay of patients with stroke (see Table 1). The patient groups differed in the number of hospital admissions across all hospital wards in the year before death. The patients with ALS and PDoed had a higher average number of hospital admissions, 2.78 and 2 times, respectively, compared to the patients with stroke, with an average of 1.46 (ischaemic) and 1.98 (haemorrhagic) admissions.

**Fig. 2:**
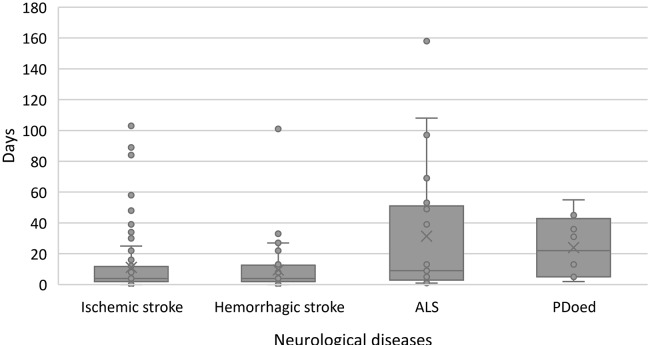
Distribution of number of days from admittance to the neurological ward until decision of end-of-life care in each patients’ group

The time from hospital admission to documenting decisions on end-of-life care varied across patient groups, as is shown in the boxplot in Fig. 2. The average time for patients with ischaemic stroke was 11.16 days (median: 4 days, range: 0-103 days), while for patients with haemorrhagic stroke, it was 9.48 days (median: 4 days, range: 0-101 days). Patients with PDoed and ALS had longer durations, with an average of 24 days (median: 22 days, range: 2–55 days) and 31.33 days (median: 9 days, range: 1-158 days), respectively.

The Cox regression results show that advanced age and ischaemic and haemorrhagic stroke diagnoses were associated with a greater likelihood of end-of-life care decisions following admission (see Table 2). Expected decision increased by increased age. Patients with haemorrhagic stroke had twice the hazard rate for end-of-life care decisions compared to patients with ALS and PDoed, while patients with ischaemic stroke had a hazard rate 1.6 times higher than patients with ALS and PDoed. Gender, marital status, and a previous treatment directive did not predict end-of-life care decision-making in patients admitted to the neurological ward.

**Table 2:** Cox regression of the hazard rate of end-of-life care decisions

Variable	Hazard rate of end-of-life care decision following admission to the neurological ward					95% CI for Exp(B)		Hazard rate of death following a decision of end-of-life care					95% CI for Exp(B)	
	B	SE	Wald	Sign	Exp (B)	Lower	Upper	B	SE	Wald	Sign	Exp (B)	Lower	Upper
Age	0.023**	0.008	7.807	0.005	1.023	1.007	1.039	-0.001	0.007	0.043	0.836	0.999	0.985	1.012
Gender (1 = male)	0.062	0.172	0.130	0.719	1.064	0.760	1.489	-0.075	0.172	0.189	0.664	0.928	0.663	1.300
Married/cohabiting^a^	0.024	0.179	0.018	0.894	1.024	0.721	1.455	0.238	0.176	1.835	0.176	1.269	0.899	1.790
Ischaemic stroke^b^	0.498**	0.237	4.417	0.036	1.645	1.034	2.618	-0.079	0.229	0.119	0.730	0.924	0.590	1.447
Haemorrhagic stroke^b^	0.714**	0.263	7.358	0.007	2.043	1.219	3.423	-0.171	0.260	0.434	0.510	0.843	0.506	1.403
Treatment directives	-0.210	0.192	1.198	0.274	0.810	0.556	1.181	0.110	0.204	0.290	0.590	1.116	0.748	1.665

**p < 0.01

a) Married/cohabiting as reference variable

b) For medical diagnoses, ALS/PDoed as a reference variable

**Fig. 3:**
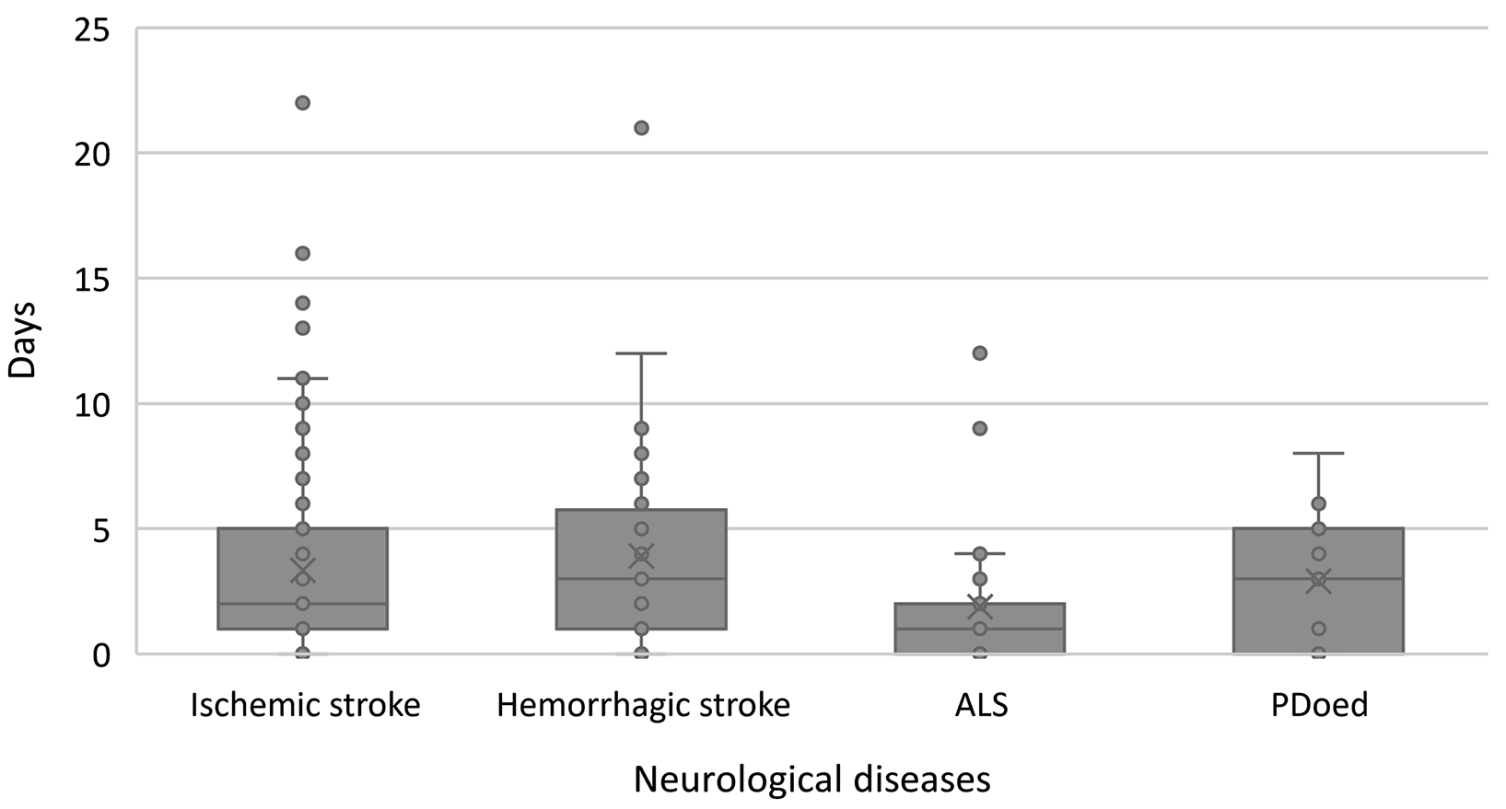
Distribution of number of days from the decision of end-of-life care until death in each patients’ group

As for the number of days from the end-of-life care decision until the patients’ death, half of the patients (*n* = 98) died within the first two days after a treatment directive of end-of-life care was initiated. The maximum period of end-of-life care was 22 days. Patients with ischaemic stroke lived for an average of 3.33 days (median: 2 days, range: 0–22 days) after the decision was made, while patients with haemorrhagic stroke lived for an average of 3.90 days (median: 3 days, range: 0–21 days) (see Fig. 3). In contrast, patients with ALS had the shortest survival time, with an average of 1.89 days (median: 1 day, range: 0–12 days), and patients with PDoed survived an average of 2.91 days (median: 3 days, range: 0–8 days) after the decision on end-of-life care. The Cox regression results showed no differences in the hazard rate following end-of-life care decision-making by the presence of a previous treatment directive, medical diagnosis, or demographic variables (see Table 2).

## Discussion

In this study, we analysed the timeframe of end-of-life care decision-making and death of patients in an acute neurological hospital ward. Our findings provide new insight into the notably short time from the point of documenting an end-of-life treatment directive to patients’ death for all patient groups and a relatively long time from admission to end-of-life care decision-making in patients with ALS and PDoed. The time from end-of-life care decision-making to death was a medium of two days for patients with haemorrhagic stroke and three days for patients with ischaemic stroke. For patients with ALS, the median was one day, and for patients with PDoed, the median was three days. This timeframe is longer than found in a study of patients with PDoed (*n* = 8), where the timeframe was 0–1 days [43]. For patients with stroke our findings are similar to those of Kim et al. (*N* = 2,721) [12] who found a median time of three days. The median time from admission to end-of-life care decision-making was four days for patients with stroke, and 9 and 22 days for patients with ALS and PDoed, respectively, e.g., patients with stroke were transitioned twice as early as patients with ALS and PDoed. Similarly, Hausammann et al. [41] and Helvig et al. [42] found a median time of three days for stroke patients and Bhansali et al. [43] found that patients with Parkinson’s disease transitioned with a median time of two days after admission, which is markedly shorter than found in our study. Comparable studies for patients with ALS were not retrieved.

Unlike patients with stroke, patients with ALS and PDoed are typically diagnosed months or years prior to their death; thus, careful planning and initiation of timely end-of-life care should be possible [30]. A major findings of this study is the distinct difference between transition to end-of-life care of patients with acute diseases (i.e., stroke) and progressive diseases (i.e., ALS and PDoed). Patients with stroke present with sudden symptoms and typically require heightened acute and intensive medical care during the initial stage of hospitalisation for which short end-of-life care might be expected [41, 42] and a long deliberation about end-of-life decision-making not possible [38]. Differently, however, in our study patients with the progressive diseases ALS and PDoed had the shortest time from end-of-life care decision-making to death.

Palliative clinical guidelines, including transition to end-of-life care, were originally developed for patients with cancer and have later been adapted for utilisation for patients with other incurable diseases [4, 9]. Different from their mandate, the very late decision-making for end-of-life care found in this study indicates that there might be shortcomings of using these guidelines unaltered for patients with neurological diseases. Also, the unpredictable and sharp decline in health in the few days before death that is reported in patients with neurological diseases [15, 23, 25–29, 32, 33] suggests that different clinical guidelines are needed for patients of different neurological disease groups [27, 30, 31, 37], containing variability in treatments within the same disease group, e.g., patients with stroke (haemorrhagic versus ischaemic stroke) [16, 22, 24, 38–42].

Patients in this study were recruited from one specialised neurological hospital ward which serves patients with a diversity of neurological diseases. This may confuse treatment goals because within the same ward there are highly acute patients in need of specialised medical treatment at the same time as there are patients who have had a long disease trajectory for whom foreseeing possible end-of-life should be conceivable [7, 30]. Making a clear distinction in the organisation and delivery of daily care between different groups of patients within the same ward might lead to better identification of unreversible signs and symptoms of dying. Therefore, segregating patients by disease groups, i.e., separating patients with stroke who could benefit from highly acute and intensive care [24, 31], from patients with ALS and PDoed who might benefit from a long-term palliative care perspective [3], might be worthwhile.

This study has generated insights into the transition to end-of-life care in patients with neurological diseases in an acute hospital ward with a mixed patient population. The decision-making process for end-of-life care for these patients should vary based on the underlying symptomatology and symptom progression of patients in different diagnostic groups. The findings highlight the need for early proactive and ongoing discussions around end-of-life care [17, 30], particularly for patients with ALS [5] and patients with PDoed [3] for whom timely planning and decision-making should be possible. There is a pressing need to assist healthcare professionals who care for patients with neurological diseases to engage in decision-making earlier in the palliative and end-of-life care process and to recognise differences between the patient groups [30].

### Strengths and limitations

A major strength of this study is that all eligible patients who died in the neurological ward from January 2011 to August 2020 were included in the study, and they were representative of patients with stroke, ALS, and PDoed in the country. We excluded patients with a short hospital stay (few hours up to 6 days) for whom end-of-life decision-making would be unrealistic. This study also has limitations. The study is retrospective, and the data had already been gathered in PHRs, which limited the research questions that could be asked. However, the PHRs provided information about real-life clinical work in an acute ward and the temporal order of study variables. A limitation is the grouping of different patient groups in the data analysis, due to small group sizes, particularly patients with Parkinson’s disease and other extrapyramidal diseases.

The short average time between the decision of end-of-life care and actual death, together with a limited sample size, may have contributed to the lack of significant differences in the study’s Cox regression comparison between different groups of patients based on demographic and diagnostic factors (Table 2). Nevertheless, this study suggests that patients with different neurological diseases receive similar end-of-life care pathways in acute wards once the end-of-life decision is finally made, irrespective of their demographic or diagnostic profile.

## Conclusions

This study highlights the notably late transition to end-of-life care in different groups of patients with neurological diseases. Understanding group differences is essential for proper end-of-life care for neurological patients. Healthcare professionals need to realize different disease trajectories of neurological diseases and develop relevant clinical guidelines to meet patient needs, particularly of those with long-term and progressive diseases. It is important that exacerbations and chronicity of diseases do not eclipse the need to transition to end-of-life care. Experiences, attitudes, and practices among neurological healthcare professionals towards end-of-life care decision-making warrant further investigation.

## Data Availability

No datasets were generated or analysed during the current study.

## Abbreviations

ALS: Amyotrophic Lateral Sclerosis
PDoed: Parkinson’s Disease and other extrapyramidal diseases
PHR: Patient Health Records
SPSS: Statistical Package for the Social Sciences
